# Pulmonary Artery Calcification in a 57-Year-Old Man

**DOI:** 10.1016/j.chest.2024.02.022

**Published:** 2024-06-07

**Authors:** Lisa Hauptmann, Johannes Ruhe, Anna Xylander, Angelina Autsch, Rene Aschenbach, Gunter Wolf, Martin Busch

**Affiliations:** a Department of Internal Medicine III, Nephrology, University Hospital Jena, Jena, Germany; b Forensic Medicine, Section of Pathology, University Hospital Jena, Jena, Germany; c Forensic Medicine, University Hospital Jena, Jena, Germany; d Institute for Diagnostic and Interventional Radiology, University Hospital Jena, Jena, Germany

## Abstract

A 57-year-old man was admitted to our hospital via the ED presenting in reduced general condition because of an infection of unknown origin, generalized edema, and dyspnea at rest (peripheral capillary oxygen saturation, 89%) that required 2 L/min intranasal oxygen. Anamnesis was complicated by an infection-triggered delirium, but his wife reported an increasing physical decay that had led to bed confinement. The BP was reduced at 88/55 mm Hg with a normal heart rate of 86 beats/min. Lung auscultation showed mild bipulmonal rales. Previous comorbidities were a BMI of 42 kg/m^2^, an insulin-dependent type 2 diabetes mellitus with a severe diabetes-related chronic kidney disease stage G4A3, and systemic arterial hypertension.

Two years ago, the patient experienced severe COVID-19 pneumonia that necessitated temporary artificial respiration with successful weaning after 2 months. Current blood count showed a leukocytosis of 12.7 × 10^9^/L and a normochromic, normocytic anemia (hemoglobin, 6.6 mM). C-reactive protein was highly increased at 236 mg/L. Serum creatinine was at 323 μmol/L (estimated glomerular filtration rate 17 mL/min/1.73 m^2^) with a proteinuria of 2,089 mg/g creatinine. Notably, serum calcium increased to 3.22 mM. The long-term medication included atorvastatin (10 mg/d), candesartan (8 mg/d), chlorthalidone (25 mg/d), febuxostat (80 mg/d), nebivolol (5 mg/d), pregabalin (25 mg/d), dulaglutide (1.5 mg/d), insulin detemir (18 units per day), and insulin lispro (70 units per day). A chest CT scan showed peribronchial pneumonic infiltrates in the right middle lobe (Fig 1) alongside previously known parenchymal changes in the lower lobes after COVID-19 pneumonia in 2020 (Fig 2). In addition, enlarged pulmonary veins in both basal lobes in comparison with the adjacent bronchus could be identified (Fig 1).

**Figure 1:**
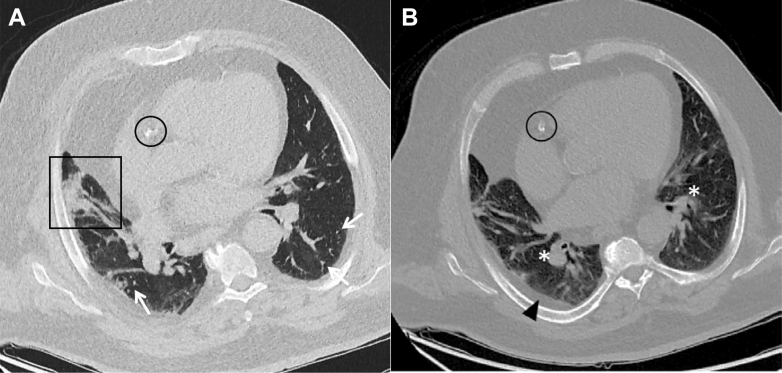
A, B, Chest CT scan on admission shows A, a focal area of subsegmental consolidation with visible air bronchogram in the middle lobe (boxed area) compatible with beginning bronchopneumonia. Furthermore, nodular hyperdensities (arrows) in the peripheral lung parenchyma are visible, potentially reflecting vascular calcifications. B, Scan additionally shows signs of pulmonary venous congestion with enlarged diameter of pulmonary veins in comparison to adjacent bronchus on both sides (asterisk) and a right-sided pleural fat deposition dorsobasal (closed triangle). Both images show minimal coronary calcification (circled area).

**Figure 2:**
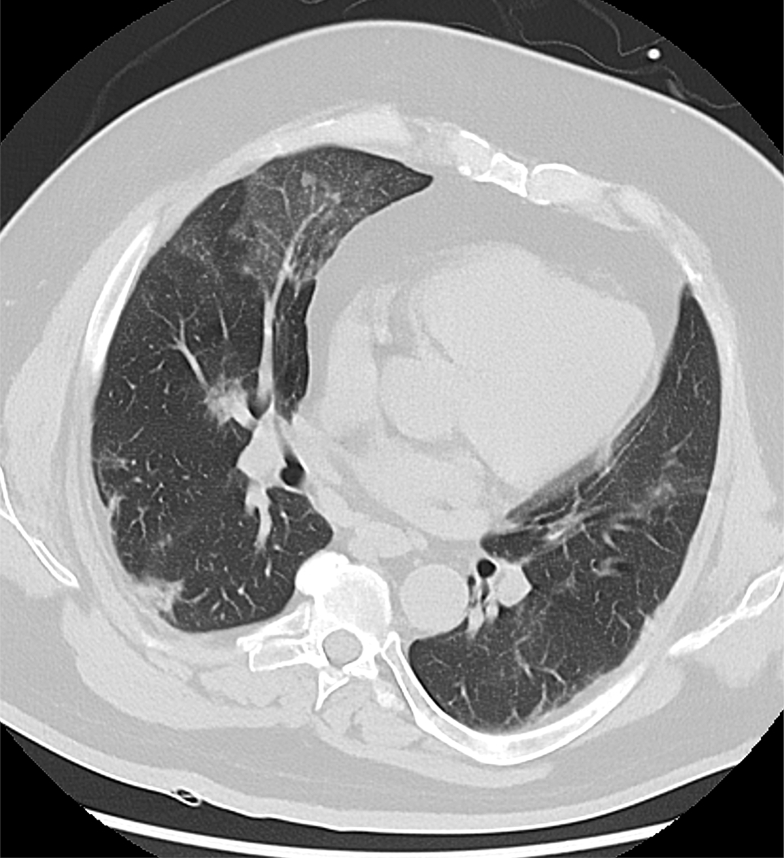
Parenchymal changes in the right middle and both lower lobes suggest COVID-19-pneumonia (previous CT scan from 2020).

A discrete leukocyturia (47/μL) and hematuria (erythrocytes, 115/μL) was seen in urinary status. Several days earlier, the patient’s general practitioner clinically suspected a urinary tract infection and initiated an oral antibiotic treatment with ciprofloxacin without previous urine culture. The urinary culture on admission remained sterile.

To exclude no response and to address the CT scan results correctly, the antibiotic treatment was altered to ceftriaxone 2g per day IV. After 4 days of inpatient therapy with initial clinical improvement in terms of reduced oxygen demand and declining delirium, the patient died suddenly and unexpectedly. An autopsy was initiated to clarify the actual cause of death.

The autopsy revealed a dilated, hypertrophic heart with an organ weight of 646 g (Fig 3). There was thickening of both left and right ventricular wall with a diameter of 2.0 cm left and 0.5 cm right. All heart valves were dilated slightly and showed the following circumferences: tricuspid valve, 12.5 cm; pulmonary valve, 8.8 cm; mitral valve, 12.0 cm; and aortic valve, 8.0 cm. In addition, the patient experienced mitral valve sclerosis. Despite moderate-to-severe coronary artery disease, neither acute nor older myocardial infarctions could be detected. Signs of right-sided heart failure were seen that included pleural effusions, congestive hepatopathy, and splenic hypertrophy.

**Figure 3:**
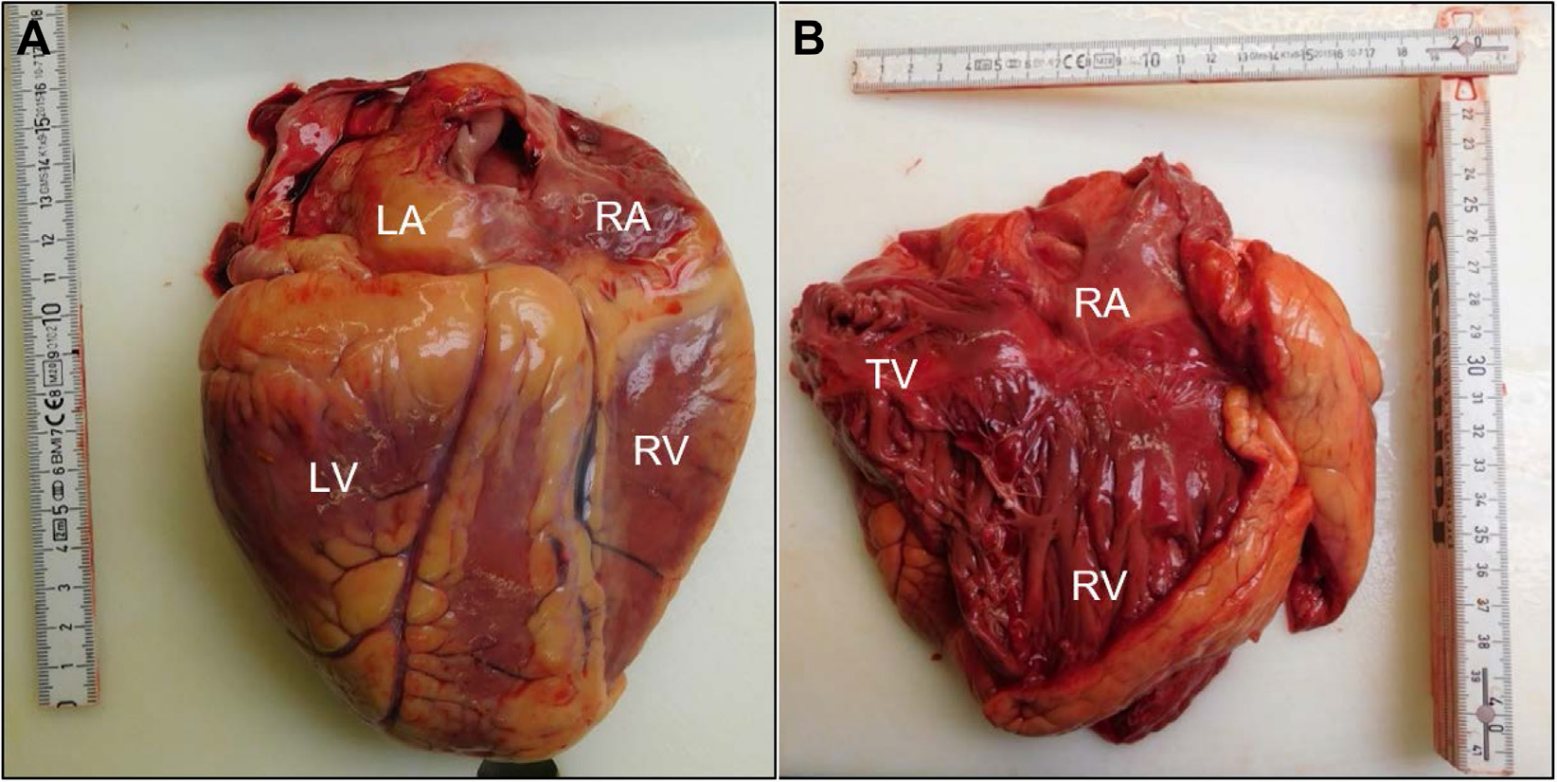
A, B, Macroscopic images of the patient’s heart show A, dilated, hypertrophic heart, view from posterior; and B, opened right atrium and ventricle with dilated tricuspid valve. LA = left atrium; LV = left ventricle; RA = right atrium; RV = right ventricle; TV = tricuspid valve

In the histologic examination of the lung, obliterating calcification patterns of arterioles and venules were detected (Fig 4).

**Figure 4:**
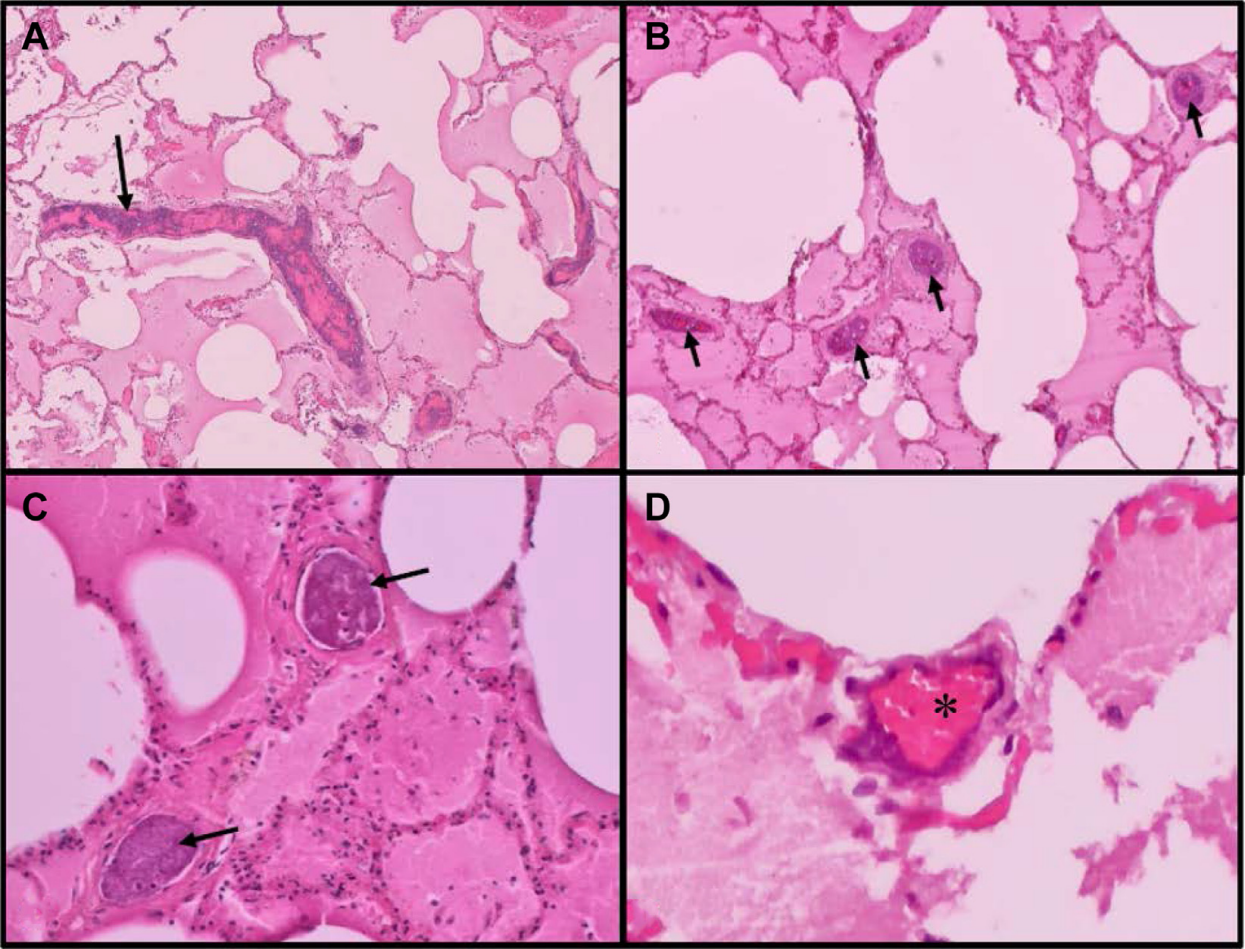
A-D, Microscopic images from the lung tissue. A, Lime precipitates (arrow) along the pulmonary vessels (original magnification, ×40). B, Smaller vessels with significant stenosis caused by calcification (arrows; original magnification, ×40). C, Higher magnitude shows obliteration of pulmonary arterioles by calcification (arrows; original magnification, ×100). D, Intramural lime precipitates with remaining intraluminal blood flow (asterisk) in a pulmonary venule (original magnification, ×200). All images: hematoxylin-eosin stain.

In the thyroid gland, the autopsy revealed a multinodular goiter, especially of the left lobe. Furthermore, a single, encapsulated nodule with a diameter of 2.5 cm was found (Fig 5A). The mass was attached to the left lobe of the thyroid gland and showed a nodular, inhomogeneous red and white cross section. Because of the location and morphologic condition (dense parathyroid tissue with dominance of chief cells and a significant lack of adipocytes) (Fig 5B), it was considered suspicious for a malignant neoplasia of the parathyroid gland.

**Figure 5:**
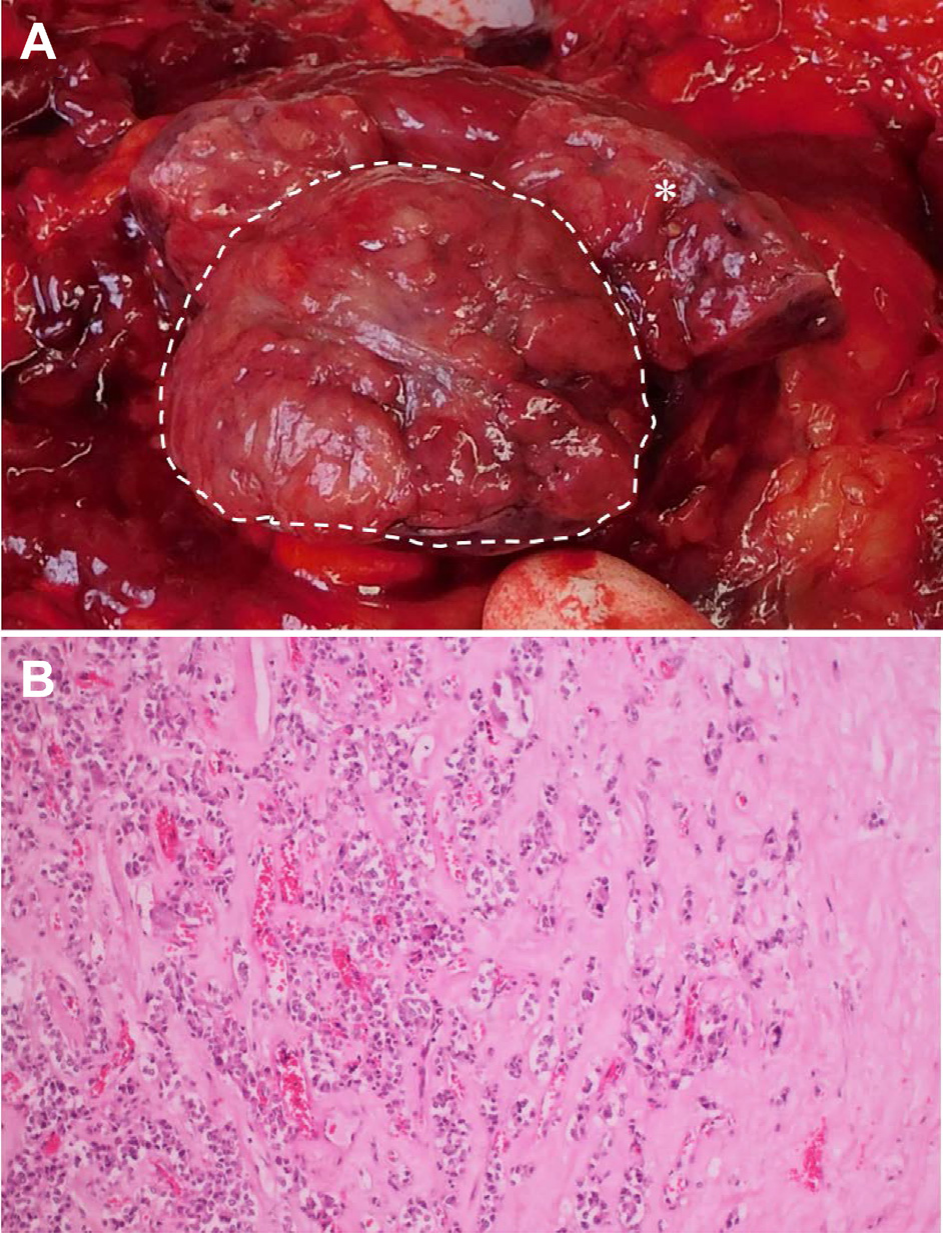
A, Left lobe of the thyroid gland (asterisk) with parathyroid neoplasia (dashed line). B, Nodular lesion near the thyroid gland with dominance of chief cells. Notice the hyalinized matrix, disorganization of the parenchyma, and lack of adipocytes (original magnification, ×100; hematoxylin-eosin stain).

*What is the diagnosis?*

*Diagnosis*: Calcification of the pulmonary vessels most likely caused by secondary pulmonary hypertension caused by severe primary hyperparathyroidism

## Discussion

### Clinical Discussion

The postmortem diagnosis of a primary hyperparathyroidism (HPT) that led to pulmonary vessel calcification with pulmonary hypertension (PH) was clinically supported by respiratory failure, hypotonia, hypercalcemia (3.22 mM; ionized calcium, 1.53 mM), and an intact (1 to 84) parathyroid hormone (iPTH) of 503 ng/L.

**Figure 6:**
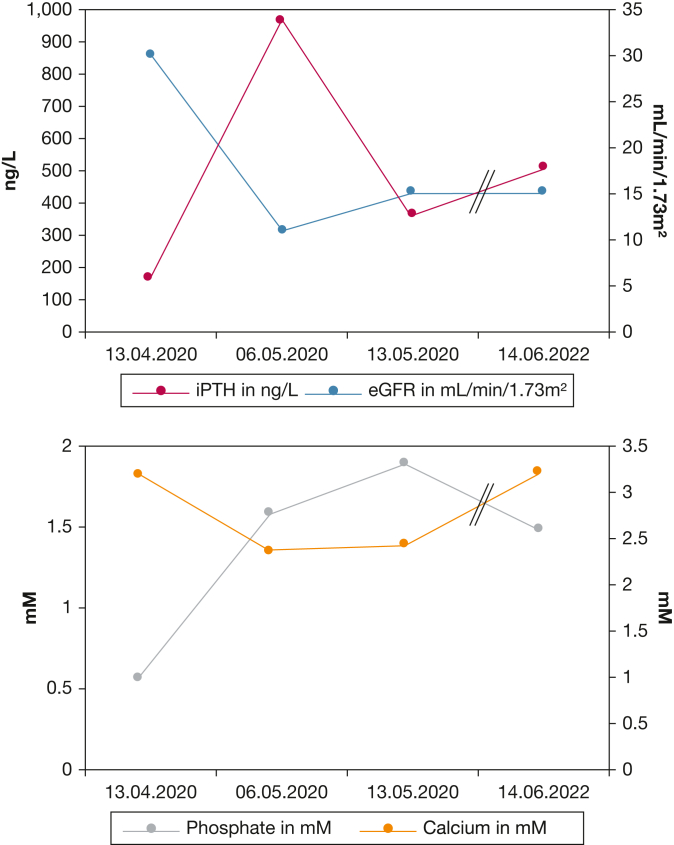
Renal function in relation to intact parathyroid hormone, calcium, and phosphate concentration. eGFR = estimated glomerular filtration rate; iPTH = intact parathyroid hormone.

**Figure 7:**
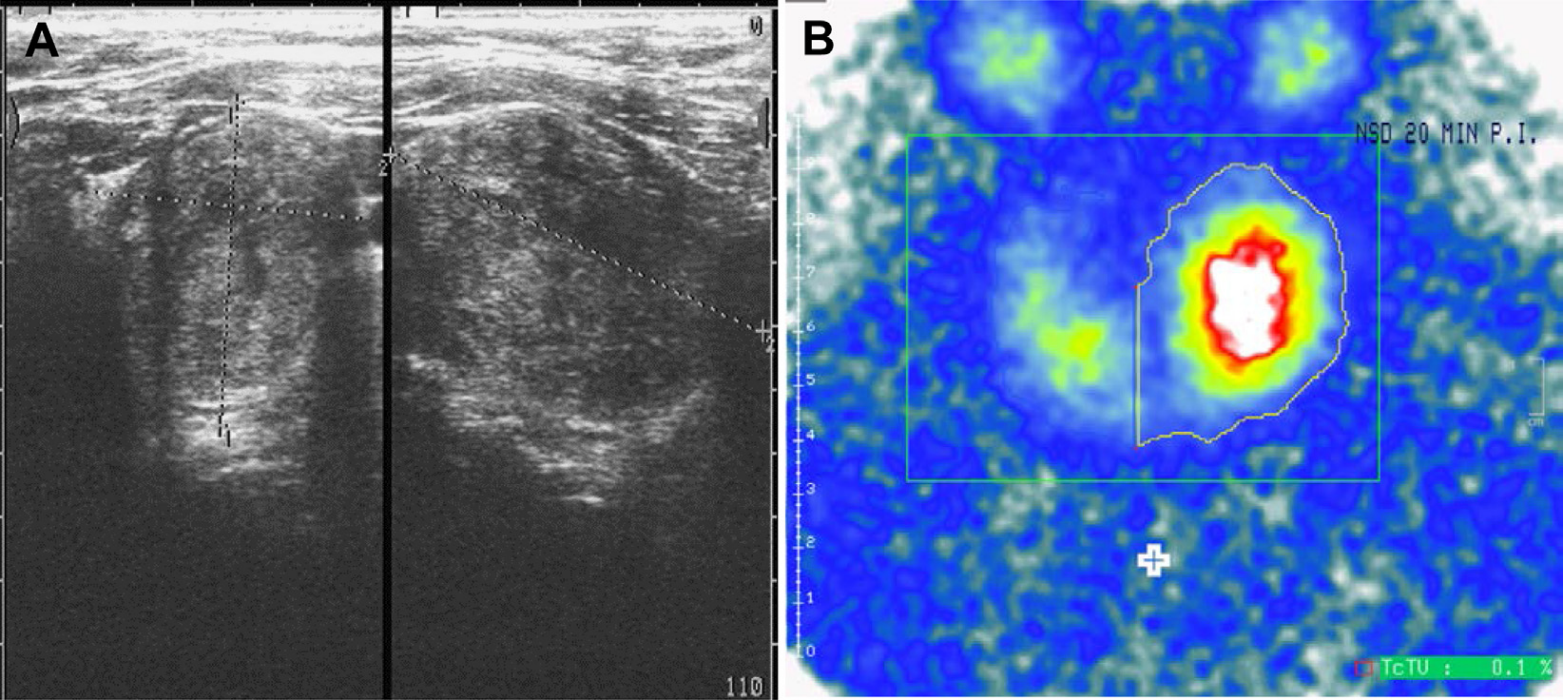
A, Sonographic finding of a nodule (approximately 2 × 3 × 4 cm) in the area of the left thyroid lobe. B, The nodule could be identified as parathyroid adenoma by technetium-99m sestamibi scintigraphy that shows intense tracer uptake in the area of lower left lobe of the thyroid. The other three parathyroid glands are colored green.

In April 2020, before being hospitalized for COVID-19, the patient's renal function was impaired at chronic kidney disease (CKD) stage G3bA3. The iPTH was approximately fourfold increased (165 ng/L) and associated with an elevated serum calcium concentration of 3.2 mM at this time (Fig 6). CKD stage 3b is usually the earliest stage of kidney disease at which time secondary HPT caused by CKD manifests and thus was suspected in this patient.^1^ However, a progressively increasing iPTH as seen in this patient (Fig 6) is rather unusual for CKD-related secondary HPT. Therefore, primary HPT was discussed, which was further supported by an inhomogeneous, echo-attenuated nodule around the left thyroid lobe that could be detected sonographically in April 2020 and subsequently could be confirmed as a single adenoma of the lower left parathyroid gland by technetium-99m sestamibi scintigraphy (Fig 7). Despite the recommendation for a parathyroidectomy, the patient refused surgery. Instead, an oral therapy with calcimimetics (cinacalcet, 60 mg/d) had to be initiated and effectively normalized serum calcium values (Fig 6). During the patient’s severe COVID-19 pneumonia in May 2020, the kidney function was further impaired, and cinacalcet was discontinued during intensive care treatment. Consequently, iPTH was at 966 ng/L in May 2020 (Fig 6). Noticing this iPTH rebound, cinacalcet was readministered, which led to a better control of iPTH. However, after hospital discharge and rehabilitation, the patient refused to take cinacalcet after July 2020.

We assume that, with a high probability, the iPTH concentrations were constantly high during the following 2 years (503 ng/L in June 2022 with increased serum concentrations of calcium [3.22 mM] and phosphate [1.48 mM]). The elevated phosphate starting from May 2020 (Fig 6) does not correspond to the pathophysiologic condition of a primary HPT. It must be assumed that phosphate excretion was limited because of CKD and/or cinacalcet therapy.

Primary HPT is an expression of autonomous hyperfunction of the parathyroid gland that leads to intensified tubular calcium reabsorption, phosphate excretion, and synthesis of 1.25-dihydroxyvitamin D. Because nephrolithiasis, nephrocalcinosis, ulcer disease, or bone pain are consequences of chronic hypercalcemia, patients with primary HPT should be evaluated for renal calcifications at the time of diagnosis.^2^ In addition, patients with primary HPT are at outstanding risk for coronary artery calcification.^3^ Because the patient had obesity and type 2 diabetes mellitus, insulin resistance might also have contributed to vascular calcification.^4^

With the use of the calcimimetic effects of cinacalcet, serum calcium can be lowered; however, in primary HPT, cinacalcet should be administered mainly to inoperable patients or as bridging therapy.^5^ The causal treatment of primary HPT is a (sub)total parathyroidectomy.

The postmortal findings in the case presented here that show obliterating calcification of small pulmonary vessels, enlargement of the right side of the heart with cardiac wall hypertrophy, and congestion of liver and spleen together with the HPT suggest the presence of PH. Because the patient’s dyspnea was sufficiently explainable by pneumonic infiltrates in a predamaged lung, only bedside echocardiography was performed during the hospital stay without estimation of the pulmonary arterial pressure.

PH is defined by a mean pulmonary artery pressure > 20 mm Hg.^6^ Although PH per se can induce significant vascular remodelling,^7^ the overall findings in this patient suggest that the persisting primary HPT caused pulmonary vessel calcifications that led to PH. It was shown previously that, not only calcification in the pulmonary vessels, but also the parathyroid hormone itself leads to increased vascular resistance that is mediated among others by the activation of vascular endothelial growth factor.^8^

The Nice Classification is used to classify PH according to its cause.^9^ This case of PH is at best reflected by class V; however, primary HPT would be a novel entity within the point of unclear multifactorial mechanisms or metabolic causes, respectively.

### Radiologic Discussion

High resolution CT scan is the modality of choice to confirm acute pulmonary damage based on preexisting pulmonary changes, especially in the setting of PH.

For the diagnosis of PH, the finding of enlarged pulmonary artery diameters, both central and peripherally, defined as an enlargement in three of four pulmonary segments compared with the corresponding airway diameter is usually demanded. However, in our case, no changes in arterial diameters could be found. Instead, we found elevated diameters of pulmonary veins in comparison with the adjacent bronchus (eg, in the peripheral lung segments) (Fig 1). Image interpretation must consider the impact of initial volume overload that lead to pulmonary venous congestion and the placing of the patient during the scan. A side-dominant rotation can lead to misinterpretation because of gravitational changes or effects of blood content in the vessels. The situation here was complicated by beginning bronchopneumonia (Fig 1), which is a finding that can be detected only by a CT scan, if the precondition of COVID-19 associated changes must be considered. The typical web-like subpleural changes caused by COVID-19 was seen here as relatively unchanged compared with 2020 (Fig 2). No macroscopic calcifications of pulmonary vessels were described in the initial assessment of the chest CT scan. This could be attributed to the presence of scattered calcification, particularly in the peripheral regions, in which case the vessel diameter is relatively small. Furthermore, a diagnosis of calcification was not the primary focus in the emergency situation. Subsequently and considering the autopsy findings, the CT scan was reevaluated. Because of this, discrete peripheral pulmonary hyperdense changes could be detected (Fig 1), which can be interpreted possibly as signs of metastatic pulmonary calcification comparable with CT scan findings, as recently described in a case series.^10^ Moreover, coronary calcification was visible in the CT scan but was considered to be mild.

In the setting of suspected primary HPT, the application of contrast media in CT scans or MRI is mandatory for the detection of hypervascular adenomas. Usually, parathyroid adenomas show hyperenhancement compared with lymph nodes.^11^ At first presentation, adenomas of the thyroid gland are detected mostly by ultrasound scanning as in this present case (Fig 7). The typical enhancement in technetium-99m sestamibi scintigraphy supported the diagnosis of a parathyroid adenoma and its localization. Further diagnostic approaches such as 4-D CT scans or various PET/CT scanning procedures were not required in this present case.

### Pathologic Discussion

The lung tissue showed aspects of dystelectatic changes, thickened alveolar septae focally with confluent fibrosis, and hyperemia with remnants of alveolar edema. However, representative specimens contained multiple small and intermediate pulmonary vessels with associated calcification and focal ossification. These calcifications were found predominantly luminal in pulmonary arterioles that led partly to a complete obliteration, whereas venules showed intramural lime precipitates (Fig 4). Because histologic sections are taken from exemplary lung sections, it is not possible to draw precise conclusions about the overall pulmonary vessel calcification distribution pattern.

Although asphyxia must be accounted for as the final cause of death, and, in particular, if the obesity, initial hypervolemia, and acute pulmonary infection are considered, the following causality was believed to have contributed significantly to his death.

Because the nodular changes of the parathyroid gland could be identified as adenomatous parathyroid neoplasms (Fig 5), the diagnosis of primary hyperparathyroidism was confirmed. Because of the parathyroid adenomas, recurrent or persistent hypercalcemia triggered progressive vascular calcification. This phenomenon was still supported by progressive CKD, in which case a loss of calcification inhibitors is evident.^12^ In addition to vascular damage caused by his other comorbidities (type 2 diabetes mellitus, obesity, arterial hypertension, previous COVID-19 infection), this likely led to the calcification of the pulmonary vessels and subsequently to an increase in pulmonary arterial pressure and PH that was to be diagnosed based on the autopsy findings. As a result and considering the volume overload with right heart dilation, progressive right-sided heart failure might have developed as the final cause of death. Thus, the untreated primary HPT presumably was causal for the progressive calcification of the pulmonary vessels and was the leading cause of death.

## Conclusions

This is, to the best of our knowledge, the first report of an obliterating calcification of small lung vessels caused by primary HPT most likely being the leading cause of death. In this context, postmortal signs of chronic and now acutely decompensated PH were evident. This exceptional case offers the opportunity to add HPT as a potential endocrinologic cause of PH to the Nice Classification of PH providing a new diagnosis and treatment option.

## Financial/Nonfinancial Disclosures

None declared.
